# Injectable and Oral Contraceptive Use and Cancers of the Breast, Cervix, Ovary, and Endometrium in Black South African Women: Case–Control Study

**DOI:** 10.1371/journal.pmed.1001182

**Published:** 2012-03-06

**Authors:** Margaret Urban, Emily Banks, Sam Egger, Karen Canfell, Dianne O'Connell, Valerie Beral, Freddy Sitas

**Affiliations:** 1 NHLS/MRC Cancer Epidemiology Research Group, National Health Laboratory Services, Johannesburg, South Africa; 2 Faculty of Health Sciences, University of the Witwatersrand, Johannesburg, South Africa; 3 National Centre for Epidemiology and Population Health, The Australian National University, Canberra, Australia; 4 The Sax Institute, Sydney, Australia; 5 Cancer Council New South Wales, Sydney, Australia; 6 Sydney Medical School—Public Health, University of Sydney, Sydney, Australia; 7 School of Public Health and Community Medicine, University of New South Wales, Sydney, Australia; 8 School of Medicine and Public Health, University of Newcastle, Newcastle, Australia; 9 Cancer Epidemiology Unit, University of Oxford, Oxford, United Kingdom; McGill University, Canada

## Abstract

**Background:**

Oral contraceptives are known to influence the risk of cancers of the female reproductive system. Evidence regarding the relationship between injectable contraceptives and these cancers is limited, especially in black South Africans, among whom injectable contraceptives are used more commonly than oral contraceptives.

**Methods and Findings:**

We analysed data from a South African hospital-based case–control study of black females aged 18–79 y, comparing self-reported contraceptive use in patients with breast (*n* = 1,664), cervical (*n* = 2,182), ovarian (*n* = 182), and endometrial (*n* = 182) cancer, with self-reported contraceptive use in 1,492 control patients diagnosed with cancers with no known relationship to hormonal contraceptive use. We adjusted for potential confounding factors, including age, calendar year of diagnosis, education, smoking, alcohol, parity/age at first birth, and number of sexual partners. Among controls, 26% had used injectable and 20% had used oral contraceptives. For current and more recent users versus never users of oral or injectable contraceptives, the odds ratios (ORs) for breast cancer were significantly increased in users of oral and/or injectable contraceptives (OR 1.66, 95% CI 1.28–2.16, *p*<0.001) and separately among those exclusively using oral (1.57, 1.03–2.40, *p* = 0.04) and exclusively using injectable (OR 1.83, 1.31–2.55, *p*<0.001) contraceptives; corresponding ORs for cervical cancer were 1.38 (1.08–1.77, *p* = 0.01), 1.01 (0.66–1.56, *p* = 0.96), and 1.58 (1.16–2.15, *p* = 0.004). There was no significant increase in breast or cervical cancer risk among women ceasing hormonal contraceptive use ≥10 y previously (*p* = 0.3 and *p* = 0.9, respectively). For durations of use ≥5 y versus never use, the ORs of ovarian cancer were 0.60 (0.36–0.99, *p* = 0.04) for oral and/or injectable contraceptive use and 0.07 (0.01–0.49, *p* = 0.008) for injectable use exclusively; corresponding ORs for endometrial cancer were 0.44 (0.22–0.86, *p* = 0.02) and 0.36 (0.11–1.26, *p* = 0.1).

**Conclusions:**

In this study, use of oral and of injectable hormonal contraceptives was associated with a transiently increased risk of breast and cervical cancer and, for long durations of use, with a reduced risk of ovarian and endometrial cancer. The observed effects of injectable and of oral contraceptives on cancer risk in this study did not appear to differ substantially.

*Please see later in the article for the Editors' Summary*

## Abstract 2

A case-control study conducted in South Africa provides new estimates of the risk of specific cancers of the female reproductive system associated with use of injectable and oral contraceptives.

## Editors' Summary

**Background:**

Hormonal contraceptives are among the most commonly used medications. Globally, more than 210 million women currently use either hormonal contraceptive pills or injectable contraceptives. Contraceptive pills usually contain manmade versions of the female sex hormones estrogen and progesterone (the combined oral contraceptive, or “pill”); most injectable hormonal contraceptives contain only manmade progesterone preparations. Hormonal contraceptives, which prevent pregnancy by disrupting the cyclical changes in estrogen and progesterone levels that prepare the body for pregnancy, have revolutionized birth control since they first became available in the early 1960s. However, it is now known that taking the pill also influences women's risk of developing cancers of the female reproductive system. Current and recent users have an increased risk of developing breast and cervical cancer (the cervix is the structure that connects the womb to the vagina) compared to never users, although this increased risk quickly disappears when women stop taking the pill. By contrast, women who have used the pill have a reduced risk of developing ovarian cancer and cancer of the womb (endometrial cancer) compared to never users that increases with the duration of pill use and persists for many years after use ceases. These effects on reproductive system cancers are thought to occur because these cancers depend on naturally occurring sex hormones for their development and growth.

**Why Was This Study Done?:**

Although the evidence that the pill influences the risk of developing cancers of the female reproductive system is extensive, much less is known about how injectable hormonal contraceptives affect cancer risk. In this hospital-based case–control study (a study that compares the characteristics of people with and without a specific condition), the researchers investigate the relationship between the use of oral and injectable hormonal contraceptives and cancers of the breast, cervix, ovary, and endometrium among black South African women. Injectable contraceptives have been used for longer in South Africa than elsewhere and are used more commonly than oral contraceptives among black South African women.

**What Did the Researchers Do and Find?:**

As part of the Johannesburg Cancer Case Control Study, which recruits black patients attending Johannesburg public referral hospitals for cancer treatment, the researchers compared hormonal contraceptive use in women with breast, cervical, ovarian, or endometrial cancer with contraceptive use in women diagnosed with other cancers such as lung, colon, and rectal cancers, which are not known to be influenced by hormonal contraceptives. Among the controls, a quarter had used injectable contraceptives and a fifth had used oral contraceptives. After adjusting for other factors that might influence cancer risk such as age, smoking, and number of sexual partners, the odds ratio (OR) of breast cancer among current and recent users of oral and/or injectable contraceptives compared to never users was 1.66. That is, the risk of developing breast cancer among current and recent users of hormonal contraceptives was 1.66 times that among never users. For women using oral contraceptives exclusively or injectable contraceptives exclusively, the ORs of breast cancer were 1.57 and 1.83, respectively. There were also increases in cervical cancer risk among current and recent users of hormonal contraceptives compared to never users, but no significant increase in breast or cervical cancer risk among women who had ceased hormonal contraceptive use more than ten years previously. Finally, the use of hormonal contraceptives for more than five years reduced the risk of both ovarian and endometrial cancer.

**What Do These Findings Mean?:**

These findings indicate that, among black women in South Africa, the use of oral or injectable hormonal contraceptives is associated with a transiently increased risk of breast and cervical cancer, and that extended use of these contraceptives is associated with a reduced risk of ovarian and endometrial cancer. Moreover, they suggest that the effects of oral versus injectable contraceptives on cancer risk do not differ substantially, although for endometrial and ovarian cancer the small number of cases exposed to injectable contraceptives limits the accuracy of the risk estimates. Other limitations of this study include the possibility that the findings may be affected by uncontrolled confounding. That is, women who used hormonal contraceptives may have shared other unidentified characteristics that affected their cancer risk. Nevertheless, these findings provide new information about the effects of oral and injectable hormonal contraceptives on cancer risk that should help women make informed decisions about their choice of contraceptive method.

**Additional Information:**

Please access these web sites via the online version of this summary at http://dx.doi.org/10.1371/journal.pmed.1001182.

- The US National Cancer Institute provides information on breast cancer (including personal stories from breast cancer survivors), cervical cancer, ovarian cancer, and endometrial cancer for patients and health professionals, and a fact sheet on oral contraceptives and cancer risk (in English and Spanish)
- Cancer Research UK also provides information on breast cancer, cervical cancer, ovarian cancer, and endometrial cancer and information about the birth control pill and cancer risk
- Eyes on the Prize, an online support group for women who have had cancers of the female reproductive system, has personal stories; further personal stories about breast, cervical, and ovarian cancer are provided by the charity Healthtalkonline

## Introduction

Hormonal contraceptives are among the most commonly used medications. Worldwide, in 2007, 9% of women aged 15–49 y were estimated to be using the oral contraceptive pill and 4% were using injectable contraceptives or implants [1], amounting to over 210 million women exposed to these contraceptive types [2].

Large-scale epidemiological evidence has shown that use of oral contraceptives significantly affects the risk of cancers of the liver and of the female reproductive system, specifically cancers of the breast, cervix uteri, ovary, and endometrium [3],[4]. Hence, the International Agency for Research on Cancer classifies combined oral contraceptives as carcinogenic to humans, concluding that there is sufficient evidence of an increased risk of breast, cervical, and liver cancer in current and recent users [3],[4]. The Agency also states that there is convincing evidence that women who have used combined oral contraceptives have a reduced risk of both ovarian and endometrial cancer [3],[4]; this reduction in risk increases with increasing duration of use and persists for many years after use ceases [3]–[5]. Furthermore, the current worldwide evidence indicates that use of combined oral contraceptives does not significantly influence the risk of any other cancers [3],[4].

Injectable contraceptives are predominantly given as progestagen-only depot preparations—notably, depot medroxyprogesterone acetate (DMPA, Depo-Provera)—although a small proportion contain both oestrogen and progestagen [6]. Despite their approval for use in over 100 countries and their widespread use since the 1960s, particularly in low income countries, evidence regarding the relationship between injectable progestagen-only contraceptives and cancer is limited [6]. The data that are available indicate no material difference in the effect on breast cancer risk between combined oral contraceptives and injectable progestagens [7]–[10], suggesting that injectable contraceptive use is likely to increase risk. However, in studies to date, the individual relative risk estimates for breast cancer in users of injectable contraceptives exclusively are not significantly increased [7]–[10]. Pooled data on cervical cancer, published in 2007, indicate an increase in risk with injectable progestagen use for 5 y or more [11]. The worldwide evidence regarding injectable progestagen-only contraceptives indicates a reduction in the risk of endometrial cancer in users [12],[13], and no significant effect on ovarian cancer [13],[14], but these findings are based on small sample sizes [12]–[14]. Moreover, since some women use both combined oral contraceptives and injectable progestagens during their lifetime, there are difficulties establishing the independent effects of injectable progestagen contraceptives on cancer risk. The most recent assessment from the International Agency for Research on Cancer was in 1999 and hence included much of the relevant data on breast, ovarian, and endometrial cancer, but not the 2007 data on cervical cancer; it concluded that there was inadequate evidence in humans for the carcinogenicity of progestagen-only contraceptives [6].

In South Africa, injectable progestagen-only contraceptives have been used more commonly, and for longer durations, than anywhere else in the world [1],[6],[15]. The current analysis was based on data from a large-scale hospital-based case–control study of all cancer types in Johannesburg, South Africa [16]–[18]. The objective was to investigate the relationship between use of oral and injectable hormonal contraceptives and cancers of the breast, cervix uteri, ovary, and endometrium.

## Methods

### Ethics Statement

The study was approved by the University of the Witwatersrand Human Research Ethics Committee (Medical).

### Overview

This study uses an established case–control design for cancer epidemiology studies done in resource-limited settings, where cases are individuals with the cancer of interest and controls are individuals with other cancers that are not associated with the exposure under investigation [16]–[19]. This approach has the advantage, in such settings, of minimising possible referral bias, which could result if controls without cancer and/or with other conditions were selected, in which case there might be underlying differences in the access to care of cases and controls (see Discussion). To guard against the possibility that some hitherto undescribed association between oral and injectable contraceptive use and a control cancer could materially influence the main results, sensitivity analyses were conducted systematically excluding specific cancer types from the control group.

### Study Sample

The Johannesburg Cancer Case Control Study is a large ongoing case–control study recruiting self-defined black (not mixed race/ancestry) male and female cancer patients with all cancer types, conducted at the greater Johannesburg public referral hospitals that offer cancer treatment. Female patients recruited from 8 March 1995 to 31 December 2006 were included in the present analysis. Trained nurses used a standard questionnaire to interview newly diagnosed black cancer patients in their preferred language (generally Zulu or Sotho). Participants were interviewed as soon as possible (maximum 6 mo) after diagnosis, prior to receiving chemotherapy and/or radiotherapy (verified from medical records). For all analyses, age at and calendar year of interview are taken as being age at and calendar year of diagnosis. Socio-demographic and behavioural information was solicited, including age, birthplace, residence, years of education, alcohol and tobacco use, reproductive history, and lifetime sexual history. In the large majority of cases the clinical diagnosis of cancer was supported by laboratory investigations giving microscopic verification. “Cases” for this study were women with a newly diagnosed invasive breast, cervical, ovarian, or endometrial cancer. Controls consisted of women diagnosed with cancer types that have no known relationship to oral or injectable contraception, based on data from the International Agency for Research on Cancer [4],[6] and the authors' knowledge of the area. Excluded from the controls were women with cancers of the liver and biliary system (*n* = 52); other genital cancers (total *n* = 213: vulva [*n* = 125], vagina [*n* = 22], placenta [*n* = 32], other [*n* = 34]); Kaposi sarcoma (*n* = 278), because of its overwhelming association with HIV and because its preponderance among women in HIV-positive individuals suggests that some hormonal factors may be at play; and cancers of ill-defined, secondary, or unspecified sites (*n* = 157), because they could not be assigned to be cases or controls.

### Classification of Use of Hormonal Contraceptives

Women were asked separately about oral and injectable contraceptives: (1) Have you ever taken them? (2) If yes, how old were you when you started taking them? (3) If yes, how old were you when you stopped taking them? (4) If yes, how long in total did you take them? Women reported their age at starting and stopping contraceptives in whole years, while the reporting of duration could include months. To account for the time that the different contraceptives take to be eliminated from a woman's body, in recording of duration of use, 1 mo was added to the date of the last prescription for oral contraceptives, and 2 or 3 mo were added to the time of the last injection for injectable contraceptives, depending on the preparation used. Responses to question 1 indicated whether the patient had ever used oral and/or injectable contraceptives. Responses to question 3 combined with age at cancer diagnosis (i.e., the approximate time of recruitment into the study) provided the number of years since last use of oral and/or injectable contraceptives.

Discrete categories were then created for users of oral contraceptives exclusively, users of injectable contraceptives exclusively, users of both oral and injectable contraceptives, and users of oral and/or injectable contraceptives. “Users of oral contraceptives exclusively” was defined as women who reported ever having used oral contraceptives but never having used injectable contraceptives. “Users of injectable contraceptives exclusively” was defined as women who reported ever having used injectable contraceptives but never having used oral contraceptives. “Users of both oral and injectable contraceptives” was defined as women who reported ever having used both oral and injectable contraceptives, and “users of oral and/or injectable contraceptives” was a combined group including women who reported having used either or both oral and injectable contraceptives. For these last two groups, time since last use was calculated as time since last reported use of either oral or injectable contraceptives, and duration of use was calculated by adding together the total amount of time that either oral or injectable use was reported. For each of the use categories, variables were then constructed as follows: ever use (ever versus never), total duration of use (never, <5 y, ≥5 y), and time since last use (never, <10 y, ≥10 y). The categories used in these variables were defined a priori and were broadly based on those used in prior analyses, for oral contraceptives, taking into account the sample size in the relevant categories [5],[7],[11].

Based on the most recent meta-analyses of the worldwide evidence, we hypothesised that recency of use of hormonal contraceptives would be most important for cancers of the breast and cervix, i.e., that increased risks would be seen in current and recent users [7],[11]. We also hypothesised, based on prior analyses for oral contraceptives, that for cancers of the ovary and endometrium, duration of use would be most important and that reductions in risk would be more likely to be seen in women who had used hormonal contraceptives for long periods of time [4],[5]. The analyses were structured to take account of these hypotheses, focusing on time since last use for analyses relating to cancers of the breast and cervix and on duration of use for cancers of the ovary and endometrium.

For this study, oral contraceptives were assumed to be combined oestrogen-progestagen oral contraceptives, as progestagen-only pills distributed in South African public sector clinics are recommended only for breastfeeding women [20]; the data collection did not distinguish between oral combined and oral progestagen-only contraceptives. Injectable contraceptives were assumed to be progestagen-only preparations. More detailed data from 111 consecutive injectable contraceptive users taking part in this study, with contraceptive use over the relevant time period, showed that 80% had used DMPA only, 12% had used norethisterone oenanthate only, and 8% had used DMPA and norethisterone oenanthate (unpublished data).

### Statistical Methods

Evidence regarding the relationship of potential confounding factors to the outcomes of interest was obtained from the relevant published evidence, e.g., [5],[21]. The distribution of responses for potential confounding variables according to use of oral and injectable contraceptives was tabulated for cases and controls. For each of the four “case” cancer types, separate multivariable unconditional logistic regression models were fitted to the data. The relationships between hormonal contraceptives and the specific cancer types were examined according to ever use, recency of use, and duration of use.

In order to investigate independently the effects of oral and injectable contraceptive use, the estimated odds ratios (ORs) for specific cancer types were presented for users of oral contraceptives exclusively, users of injectable contraceptives exclusively, users of both oral and injectable contraceptives, and users of oral and/or injectable contraceptives. Women who had never used either oral or injectable hormonal contraceptives were used as the reference group throughout. Cancer-specific multivariable unconditional binary logistic regression models were fitted to the data, and separate models were fitted for non-mutually exclusive categories of contraceptive use (e.g., “both oral and injectable” and “oral and/or injectable”).

All multivariable unconditional logistic regression models were adjusted for age at diagnosis (18–29, 30–34, 35–39,…, 75–79 y, with the two youngest age groups combined for the endometrial cancer analyses to remove a zero cell); year of diagnosis (1995, 1996,…, 2006); years in full-time education (0–4 y, 5–7 y, 8+ y); smoking status (never versus ever); alcohol use (ever versus never—in this population a substantial proportion of women were lifetime non-drinkers); parity/age at first birth (no live births, 1–3 live births/<21 y of age at first birth, >3/<21, 1–3/21+, >3/21+); area of residence (rural versus urban); province of birth (Gauteng, Limpopo, Free State, KwaZulu Natal, Mpumalanga, Northwest, Eastern Cape, other); and lifetime number of sexual partners (0–1, 2–5, 6+) (0 and 1 sexual partners were combined into one category because only 14 women had reported having had no sexual partners). Wald tests for heterogeneity and corresponding *p*-values—referred to here as *p*(heterogeneity)—were used to compare the associations between oral contraceptive and injectable contraceptive use and risk of specific cancers, and to examine effects according to duration of use and time since last use.

HIV infection is a potential confounder in the relationship between hormonal contraceptives and cervical cancer; data on HIV status, based on serological testing of blood collected at the time of interview, was available for 90% of study participants. We conducted sensitivity analyses of the effect of additional adjustment for HIV status in this relationship. However, because additional adjustment for HIV did not materially affect the OR estimates for hormonal contraceptives and cervical cancer (see Results), and because HIV infection is not considered a potential confounder in the relationship between hormonal contraceptives and breast, ovarian, and endometrial cancers, HIV status was not included in the primary analysis models.

The estimated ORs for cervical cancer were not adjusted for history of cervical cancer screening (Pap smears) because this item was added to the study questionnaire in 2001 and is missing for 61% of participants. We did, however, conduct sensitivity analyses using the reduced complete-case datasets whilst additionally adjusting for cervical smear history in the analysis of cervical cancer, and we also performed analyses on the full dataset after multiple imputation of the unobserved Pap smear histories. Briefly, this involved imputing missing Pap smear histories (“never/this illness only” versus “yes before this illness”) of cervical cancer and control patients 20 times using a binary logistic imputation model. Predictors in the imputation model included all confounders included in the primary analysis models, time since last contraceptive use (never oral or injectable, oral <10 y/never injectable, injectable <10 y/never oral, oral and injectable <10 y, oral ≥10 y/never injectable, injectable ≥10 y/never oral, oral and injectable ≥10 y) and also the participant's disease status (cervical cancer case or control). The analysis models for the multiply imputed data contained the same covariates as those in the corresponding primary analysis models with additional adjustment for Pap smear history (“never/this illness only” versus “yes before this illness”).

The effect of systematically excluding each control cancer type, in turn, from the control group on the estimated OR for the main exposure–outcome relationships of interest was also examined.

All analyses were performed using Stata 11.0 software (StataCorp).

## Results

For the study period, there was a total of 5,702 study participants with full information on the exposures and risk factors of interest. This sample included women with newly diagnosed invasive breast (*n* = 1,664), cervical (*n* = 2,182), ovarian (*n* = 182), or endometrial (*n* = 182) cancer. There were 1,492 controls, comprising patients with other types of invasive cancers not known to be influenced by hormonal contraceptive use, as described in Methods. There were numerous cancer types among the controls, with the most common types being oesophageal cancer (*n* = 301), colon and rectal cancer (*n* = 163), non-Hodgkin lymphoma (*n* = 125), and lung cancer (*n* = 118).

Compared with women who had never used hormonal contraceptives, users were, on average, younger, more educated, and less likely to live in a rural area (Table 1). Users were less likely than non-users, on average, to have ever smoked, to have consumed alcohol, to have one or no sexual partners, and to have had more than three live births. These differences were similar when comparing users and non-users of oral contraceptives and when comparing users and non-users of injectable contraceptives.

**Table 1:** Demographic and risk factor characteristics of case and control participants, according to use of hormonal contraceptives.

Characteristic	Never Used Hormonal Contraceptives		Ever Used Oral Contraceptives, Never Used Injectable Contraceptives		Ever Used Injectable Contraceptives, Never Used Oral Contraceptives		Ever Used Oral and/or Injectable Contraceptives	
	Cases (*n* = 2,365)	Controls (*n* = 946)	Cases (*n* = 537)	Controls (*n* = 156)	Cases (*n* = 838)	Controls (*n* = 249)	Cases (*n* = 1,845)	Controls (*n* = 546)
Age	56	60	49	48	44	39	45	43
Years of education	7	7	9	9	9	9	9	9
1 or no sexual partners	413 (17.5%)	201 (21.2%)	53 (9.9%)	18 (11.5%)	76 (9.1%)	33 (13.3%)	163 (8.8%)	67 (12.3%)
Lives in rural area	567 (24.0%)	188 (19.9%)	76 (14.2%)	22 (14.1%)	155 (18.5%)	35 (14.1%)	290 (15.7%)	74 (13.6%)
Ever tobacco smoker	560 (23.7%)	293 (31.0%)	85 (15.8%)	22 (14.1%)	121 (14.4%)	52 (20.9%)	280 (15.2%)	94 (17.2%)
Ever drink alcohol	1,001 (42.3%)	418 (44.2%)	189 (35.2%)	47 (30.1%)	317 (37.8%)	97 (39.0%)	692 (37.5%)	199 (36.4%)
>3 live births	1,076 (45.5%)	453 (47.9%)	200 (37.2%)	61 (39.1%)	375 (44.7%)	79 (31.7%)	773 (41.9%)	196 (35.9%)
Aged ≥21 y at first birth	1,079 (45.6%)	432 (45.7%)	261 (48.6%)	80 (51.3%)	376 (44.9%)	111 (44.6%)	837 (45.4%)	252 (46.2%)
Born in Gauteng Province	921 (38.9%)	355 (37.5%)	277 (51.6%)	87 (55.8%)	333 (39.7%)	119 (47.8%)	833 (45.1%)	271 (49.6%)
Recruitment year 2001–2006	1,109 (46.9%)	429 (45.3%)	308 (57.4%)	93 (59.6%)	545 (65.0%)	156 (62.7%)	1,170 (63.4%)	351 (64.3%)

Age and education data are medians; all other data are *n* (percent).

Table 2 shows the distribution of hormonal contraceptive usage patterns and adjusted ORs for the specific cancer types, comparing women who had ever used hormonal contraceptives with those who had never used them. Among controls, 37% had ever used oral and/or injectable contraceptives, 20% were classified as ever users of oral contraceptives, 10% were users of oral contraceptives exclusively, 26% were ever users of injectable contraceptives, and 17% were users of injectable contraceptives exclusively. Overall, 49% and 44% of the women with breast or cervical cancer had ever used injectable and/or oral contraceptives respectively, whereas 26% of the women with ovarian cancer had used hormonal contraceptives, and 17% of women with endometrial cancer had used them.

**Table 2:** Frequencies and adjusted odds ratios for breast, cervical, ovarian, and endometrial cancer according to ever/never oral and injectable contraceptive use combinations.

Contraceptive Use	Control	Breast Cancer		Cervical Cancer		Ovarian Cancer		Endometrial Cancer	
	*n* (Percent)	*n* (Percent)	OR (95% CI)	*n* (Percent)	OR (95% CI)	*n* (Percent)	OR (95% CI)	*n* (Percent)	OR (95% CI)
Total	1,492 (100%)	1,664 (100%)		2,182 (100%)		182 (100%)		182 (100%)	
Never injectable or oral contraceptive user	946 (63%)	856 (51%)	1.00	1,223 (56%)	1.00	135 (74%)	1.00	151 (83%)	1.00
Ever oral, never injectable	156 (10%)	256 (15%)	1.28 (1.00, 1.64)	241 (11%)	0.97 (0.76, 1.24)	23 (13%)	0.88 (0.52, 1.50)	17 (9%)	1.01 (0.55, 1.85)
Ever injectable, never oral	249 (17%)	344 (21%)	1.31 (1.03, 1.65)	474 (22%)	1.23 (0.99, 1.53)	10 (5%)	0.35 (0.17, 0.71)	10 (5%)	0.69 (0.33, 1.46)
Ever injectable, ever oral	141 (9%)	208 (13%)	1.17 (0.89, 1.54)	244 (11%)	1.12 (0.86, 1.45)	14 (8%)	0.69 (0.36, 1.32)	4 (2%)	0.39 (0.13, 1.12)
Ever injectable and/or ever oral	546 (37%)	808 (49%)	1.26 (1.05, 1.52)	959 (44%)	1.12 (0.94, 1.33)	47 (26%)	0.63 (0.41, 0.97)	31 (17%)	0.75 (0.45, 1.22)

OR adjusted for age at diagnosis, year of diagnosis, education, tobacco smoking, alcohol consumption, parity/age at first birth, number of sexual partners, urban/rural residence, and province of birth, where appropriate.

The risk of breast cancer was significantly increased among women who had ever used oral and/or injectable contraceptives, compared to never users of hormonal contraceptives, and the risk of ovarian cancer was significantly reduced among such ever users (Table 2). To test our a priori hypotheses, emphasis was placed initially on time since last use of hormonal contraceptives for analyses relating to breast and cervical cancer, and on duration of use for ovarian and endometrial cancer. There were relatively few cases of cancers of the ovary and endometrium, so power was limited.

### Breast Cancer

**Figure 1:**
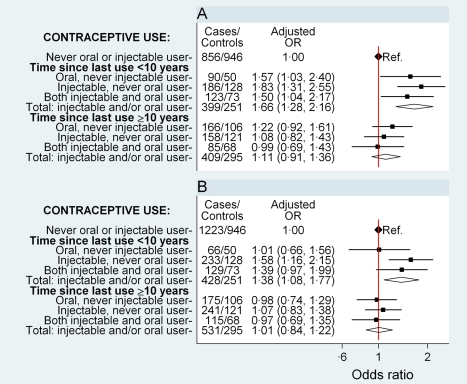
Odds ratio for breast and cervical cancer in relation to use of hormonal contraceptives, according to time since last use. Adjusted ORs (95% CI) for (A) breast cancer and (B) cervical cancer in relation to use of oral and injectable contraceptives, adjusted for age at diagnosis, year of diagnosis, education, tobacco smoking, alcohol consumption, parity/age at first birth, number of sexual partners, urban/rural residence, and province of birth. Squares represent ORs, and horizontal lines indicate 95% CI. Diamonds represent the ORs and confidence intervals for the group comprising women from all three exposure categories immediately above.

The risk of breast cancer was significantly increased (OR 1.66, 95% CI 1.28–2.16, *p*<0.001) in women who had used either oral or injectable contraceptives within the previous 10 y and did not differ significantly (OR 1.11, 0.91–1.36, *p* = 0.3) in those ceasing use ≥10 y previously, compared to women who had never used hormonal contraceptives (Figure 1A). There was no significant difference in risk between users of oral contraceptives exclusively, users of injectable contraceptives exclusively, and users of both in the last 10 y, with ORs of 1.57 (1.03–2.40), 1.83 (1.31–2.55), and 1.50 (1.04–2.17), respectively (Figure 1A; *p*[heterogeneity] = 0.6). In women who had used either or both preparations, this elevated risk declined significantly with increasing time since last use of hormonal contraceptives (*p* = 0.004) but was not significantly related to duration of use (*p* = 0.4) (Table 3).

**Table 3:** Adjusted odds ratios for breast and cervical cancer, according to time since last use and duration of use of oral and injectable contraceptives.

Contraceptive Use	Control	Breast Cancer		Cervical Cancer	
	*n* (Percent)	*n* (Percent)	OR (95% CI)	*n* (Percent)	OR (95% CI)
**Total**	1,492 (100%)	1,664 (100%)		2,182 (100%)	
**Never oral or injectable contraceptive user**	946 (63%)	856 (51%)	1.00	1,223 (56%)	1.00
**Oral and/or injectable contraceptive user**					
Time since last use <10 y overall	251 (17%)	399 (24%)	1.66 (1.28, 2.16)	428 (20%)	1.38 (1.08, 1.77)
Duration <5 y	77 (5%)	70 (4%)	1.72 (1.09, 2.70)	87 (4%)	1.43 (0.94, 2.18)
Duration ≥5 y	174 (12%)	329 (20%)	1.65 (1.26, 2.17)	341 (16%)	1.37 (1.05, 1.78)
Time since last use ≥10 y overall	295 (20%)	409 (25%)	1.11 (0.91, 1.36)	531 (24%)	1.01 (0.84, 1.22)
Duration <5 y	148 (10%)	222 (13%)	1.23 (0.96, 1.58)	286 (13%)	1.03 (0.81, 1.30)
Duration ≥5 y	147 (10%)	187 (11%)	1.00 (0.77, 1.29)	245 (11%)	1.00 (0.78, 1.27)
***p*** **(heterogeneity)—time since last use**			0.004		0.02
***p*** **(heterogeneity)—duration** a			0.4		0.96

All ORs adjusted for age at diagnosis, year of diagnosis, education, tobacco smoking, alcohol consumption, parity/age at first birth, number of sexual partners, urban/rural residence, and province of birth.

a Heterogeneity for duration <5 y versus ≥5 y, within the two categories for time since last use.

### Cervical Cancer

Compared with women who had never used hormonal contraceptives, women who had used oral and/or injectable contraceptives within the previous 10 y had a significantly elevated risk of cervical cancer (OR 1.38, 1.08–1.77, *p* = 0.01), while those ceasing use ≥10 y previously had no significant difference in risk (OR 1.01, 0.84–1.22, *p* = 0.9) (Figure 1B). The magnitude of the increase in cervical cancer risk did not differ significantly between users of oral contraceptives exclusively, users of injectable contraceptives exclusively, and users of both, in the last 10 y (*p*[heterogeneity] = 0.2); the risk in recent users of injectable contraceptives exclusively was significantly increased, compared to women who had never used hormonal contraceptives (OR 1.58, 1.16–2.15, *p* = 0.004) (Figure 1B). Risk diminished significantly with increasing time since last use of oral and/or injectable contraceptives (*p* = 0.02), but was not significantly related to duration of use (*p* = 0.96) (Table 3).

### Ovarian Cancer

**Figure 2:**
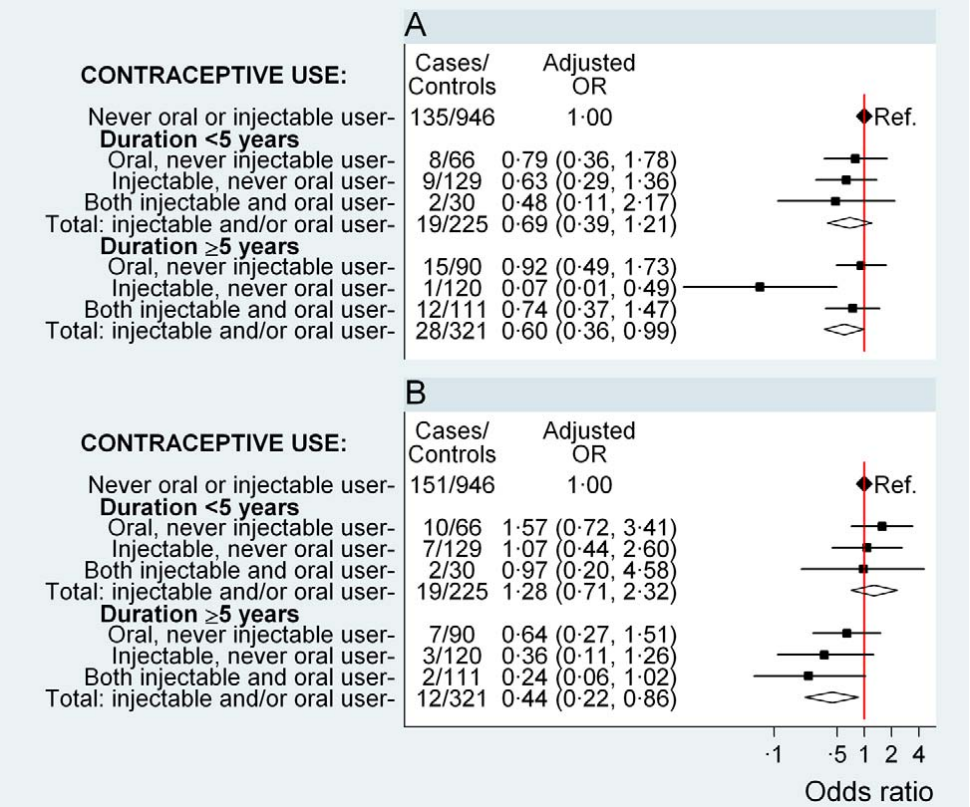
Odds ratio for ovarian and endometrial cancer in relation to use of hormonal contraceptives, according to duration of use. Adjusted OR (95% CI) for (A) ovarian cancer and (B) endometrial cancer in relation to use of oral and injectable contraceptives, adjusted for age at diagnosis, year of diagnosis, education, tobacco smoking, alcohol consumption, parity/age at first birth, number of sexual partners, urban/rural residence, and province of birth. Squares represent ORs, and horizontal lines indicate 95% CI. Diamonds represent the ORs and confidence intervals for the group comprising women from all three exposure categories immediately above.

The risk of ovarian cancer did not differ significantly between women who had never used hormonal contraceptives and those who had used oral and/or injectable contraceptives for a total of less than 5 y (OR 0.69, 0.39–1.21, *p* = 0.2) (Figure 2A). For those using hormonal contraceptives for a total of ≥5 y, the risk of ovarian cancer was significantly reduced (OR 0.60, 0.36–0.99, *p* = 0.04) (Figure 2A) compared to never users, but there was no significant difference in the overall risk of ovarian cancer by duration of use (*p* = 0.7) or by time since last use (*p* = 0.97) for oral and/or injectable contraceptives (Table 4). The sample sizes are too small for reliable comparisons by type of contraceptive used; however, significant reductions in ovarian cancer risk were observed in ever users (OR 0.35, 0.17–0.71, *p* = 0.004) (Table 2) and long-duration users of injectable contraceptives exclusively (OR 0.07, 0.01–0.49, *p* = 0.008, based on one exposed case), compared with never users of hormonal contraceptives (Figure 2A).

**Table 4:** Adjusted odds ratios for ovarian and endometrial cancer, according to duration of use and time since last use of oral and injectable contraceptives.

Contraceptive Use	Control	Ovarian Cancer		Endometrial Cancer	
	*n* (Percent)	*n* (Percent)	OR (95% CI)	*n* (Percent)	OR (95% CI)
**Total**	1,492 (100%)	182 (100%)		182 (100%)	
**Never oral or injectable contraceptive user**	946 (63%)	135 (74%)	1.00	151 (83%)	1.00
**Oral and/or injectable contraceptive user**					
Duration <5 y overall	225 (15%)	19 (10%)	0.69 (0.39, 1.21)	19 (10%)	1.28 (0.71, 2.32)
Time since last use <10 y	77 (5%)	4 (2%)	0.77 (0.22, 2.71)	0 (0%)	Insufficient data
Time since last use ≥10 y	148 (10%)	15 (8%)	0.67 (0.36, 1.23)	19 (10%)	1.39 (0.77, 2.52)
Duration ≥5 y overall	321 (22%)	28 (15%)	0.60 (0.36, 0.99)	12 (7%)	0.44 (0.22, 0.86)
Time since last use <10 y	174 (12%)	13 (7%)	0.59 (0.29, 1.18)	5 (3%)	0.51 (0.19, 1.43)
Time since last use ≥10 y	147 (10%)	15 (8%)	0.61 (0.33, 1.12)	7 (4%)	0.39 (0.17, 0.89)
***p*** **(heterogeneity)—time since last use** a			0.97		0.7
***p*** **(heterogeneity)—duration**			0.7		0.007

All ORs adjusted for age at diagnosis, year of diagnosis, education, tobacco smoking, alcohol consumption, parity/age at first birth, number of sexual partners, urban/rural residence, and province of birth.

a Heterogeneity for time since last use <10 y versus ≥10 y, within the two duration categories for ovarian cancer and within duration ≥5 y for endometrial cancer.

### Endometrial Cancer

Compared with women who had never used hormonal contraceptives, women who used oral and/or injectable contraceptives for a total of 5 y or longer had a significantly lower risk of endometrial cancer (OR 0.44, 0.22–0.86, *p* = 0.02); women who used these contraceptives for less than 5 y had no significant difference in risk (OR 1.28, 0.71–2.32, *p* = 0.4) (*p*[heterogeneity] for duration = 0.007) (Figure 2B). The sample sizes are too small to test for differences by further subdividing the data.

### Sensitivity Analyses

Additional adjustment for the number of previous Pap smears (using multiply imputed data) and for HIV status had no material effect on the OR for cervical cancer in relation to recent or past use of hormonal contraceptives (see Figures S1 and S2). Exclusion of individual cancer types from the control group had no material effect on the OR for cancers of the breast and cervix in recent and past versus never users of hormonal contraceptives, nor did it change materially the ORs for cancers of the ovary and endometrium in long- and short-duration users of hormonal contraceptives, compared with never users (see Figures S3, S4, S5, S6).

## Discussion

This study was conducted among black women in South Africa. Use of injectable contraceptives was very common, with over one-quarter of the controls in the study reporting they had used them at some point in their lives. In contrast to most of the rest of the world, use of injectable contraceptives was more common than use of oral contraceptives, and was often for long durations.

The study shows that the risk of breast cancer and cervical cancer is increased significantly among women who are current and recent users of oral and/or injectable contraceptives and, separately, among current and recent users of injectable contraceptives exclusively. The elevated risk diminishes following cessation of use, such that no significant increase in risk is present ≥10 y after ceasing use. Risk is not significantly related to duration of use and does not differ significantly between users of oral and injectable contraceptives.

There were far fewer cases of ovarian and endometrial cancer (182 cases of each, respectively) than breast and cervical cancer (about 2,000 cases of each), thus limiting statistical power. Nevertheless, we found that women who had used oral and/or injectable contraceptives for a duration of ≥5 y had significantly reduced risks of both ovarian and endometrial cancer compared with women who had never used hormonal contraceptives.

Our results show a significantly increased breast cancer risk in women who in the previous 10 y used injectable contraceptives exclusively (OR 1.83, 1.31–2.55) compared with women who have never used hormonal contraceptives, based on 186 exposed cases. These findings add substantially to the previously available evidence. The most comprehensive summary of worldwide evidence on breast cancer and use of progestagen-only injectable contraceptives was published by the Collaborative Group on Hormonal Factors in Breast Cancer in 1996 [7]. This pooled analysis found a relative risk of breast cancer of 1.17 (standard deviation = 0.13), based on 137 women with breast cancer who had used injectable progestagens within the previous 5 y, compared to never users of hormonal contraceptives [7], but this small excess risk was non-significant, and results for users of injectable contraceptives exclusively were not given [7],[22]. Another study, published in 2000 and conducted in South Africa, showed a significant increase in breast cancer risk among current users of injectable contraceptives compared to never users of hormonal contraceptives (OR 1.6, 1.1–2.3, based on 65 exposed cases), but no results were presented for current users who had exclusively used injectable contraceptives [10].

The pooled analysis on injectable contraceptive use and the risk of cervical cancer from the International Collaboration of Epidemiological Studies of Cervical Cancer [11] included data on 453 exposed cases, many of whom were also exposed to oral contraceptives. Restricting the analysis to women who had never used combined oral contraceptives, those with ≥5 y of use of injectable contraceptives had a relative risk of cervical cancer of 1.23 (1.00–1.54), compared with never users. Our finding of an OR of cervical cancer of 1.58 (1.16–2.15) among current and more recent users of injectable contraceptives (i.e., use within the past 10 y) exclusively is consistent with this, but we also found that the significant excess in risk is no longer present in women more than 10 y after their last use of injectable contraceptives.

Our finding of a significantly protective effect of the exclusive use of injectable contraceptives for ≥5 y on ovarian cancer is based on one exposed case only. However, our results are consistent with the non-significant results of the two previous studies we identified in this area, which together included only two cases exposed to injectable contraceptives exclusively [13],[14]. The most comparable study, the 1991 World Health Organization study [14], found an OR of ovarian cancer in ever users of DMPA exclusively of 0.3 (0.1–1.2) compared with never users, which is consistent with the OR of 0.35 (0.17–0.71) found here.

The sum total of the worldwide evidence regarding endometrial cancer and injectable progestagen-only contraceptives is based on ten exposed cases; it broadly indicates a reduction in risk in users but is not able to distinguish reliably the effects of injectable contraceptives independent of previous combined oral contraceptive use [12],[13],[23]. A World Health Organization study based in Thailand found an OR of endometrial cancer of 0.20 (0.03–1.63) for users of injectable contraceptives exclusively versus never users of hormonal contraceptives. However, all three cases exposed to injectable contraceptives in this study had also used oestrogens other than contraceptives pre-menopausally, so the role and magnitude of the independent effect of injectable contraceptives was unclear. A cohort study in China found a relative risk of 0.77 (0.34–1.76) in ever users versus never users of injectable contraceptives [23]. The findings of the study presented here are consistent with this evidence, with an OR of endometrial cancer of 0.36 (0.11–1.26) in long-duration users of injectable contraceptives exclusively.

The findings reported here for oral contraceptive use in relation to the risk of cancers of the breast, cervix, ovary, and endometrium are broadly compatible with the well-established relationships quantified to date [4],[7],[11],[22]. For cervical cancer, the finding of an increased risk with oral and/or injectable contraceptive use within the last 10 y is comparable to recent pooled analyses for oral contraceptive use [11]; the non-significant OR for use of oral contraceptives exclusively should be viewed in the context of the lack of a significant difference between the OR for exclusive users of oral contraceptives, exclusive users of injectable contraceptives, and users of both in the last 10 y and the fact that few women used oral contraceptives exclusively (<10%) and for long durations. In general, the patterns of risk associated with use of injectable contraceptives are similar to those seen for combined oral contraceptives.

The large numbers of women in this study, and the high prevalence of use of injectable contraceptives, means that this study is able to add to the existing evidence on the effects on cancer risk of progestagen-only injectable contraceptives: the dataset allowed us to examine risk separately in users of injectable contraceptives exclusively, particularly for breast and cervical cancer. The long duration of injectable contraceptive use in the study participants also means the risks related to prolonged use were able to be investigated. Although we were able to adjust for multiple potential confounding factors, and sensitivity analyses indicated robust findings in the face of additional adjustment, the possibility that results were affected by uncontrolled confounding cannot be excluded. Although the vast majority of the oral contraceptives investigated here are likely to be combined oral contraceptives, we cannot exclude the possibility that a small proportion comprises progestagen-only preparations. Similarly, a small proportion of the injectable contraceptives may be combined oestrogen-progestagen preparations.

The Johannesburg Cancer Case Control Study has proven valuable for investigation of other exposures and outcomes and has been conducted efficiently for over 10 y in a resource-poor and logistically difficult setting [16]–[18]. It is essentially a variant of the hospital-based case–control study design, and can also be seen as having characteristics in common with proportional mortality analyses [19],[24]. Using these methods, with controls being individuals with cancers unrelated to the exposure under investigation, the Johannesburg cancer study has successfully investigated cancer risk in relation to human herpes virus 8 [16], HIV [25], and tobacco smoking [25], producing findings that have either been replicated in later studies or are in keeping with the known evidence [26],[27]. As well as its practicality in the South African setting, the design also minimises problems associated with referral bias. In keeping with other case–control studies, self-reported exposures may be affected by recall bias; however, with this design “controls” have cancer as well, which may attenuate the problem, unless there is the perception that a specific cancer is related to contraceptive use. Although we are unable to identify any specific biases that this study design would be likely to introduce, it is not possible to exclude entirely the possibility that the findings observed here are influenced by unidentified biases or other factors. The sensitivity analyses indicate that the choice of individual cancers for inclusion in the control group did not have a meaningful impact on the main results. Furthermore, the fact that the patterns of cancer risk in relation to combined oral contraceptive use observed here are similar to those in previous studies is reassuring, and suggests that there is unlikely to be a systematic or structural problem with the study methods. The finding that the risks of breast and cervical cancer are increased and the risks of ovarian and endometrial cancer are decreased with exposure to hormonal contraceptives, using an identical set of controls, is of particular importance in this respect, since methodological problems or biases in control selection would tend to produce results skewed systematically in one direction.

This study was designed a priori to focus on black women who belong to a disadvantaged and under-researched community, but nonetheless represent around 79% of the South African population and 74% of the residents of Gauteng Province, where the study was based [28]. While certain biological factors may vary according to ethnicity, in South Africa access to services is still heavily influenced by racial factors, such that only around 7% of black individuals have medical insurance, compared to a large majority in whites and an intermediate proportion in the Asian and mixed race/ancestry populations [29]. The restriction of the study to black women and recruitment through public sector hospitals, which serve this largely uninsured group, minimises potential confounding by racial, socioeconomic, and health-services-related factors.

The hospitals that recruited for this study are the only public tertiary referral hospitals for medical oncology and radiation therapy in the study area. Current and historic referral patterns mean that the patients constituting cancer cases and cancer controls in this study should have similar probabilities of presentation at these hospitals and recruitment into the study, and this probability should not vary substantively according to exposure to hormonal contraceptives. Additional adjustment for socio-demographic factors, including education, urban/rural residence, and province of birth further safeguards against this.

The cancer profile of the participants interviewed resembled the background distribution of histologically reported cancers in black women in South Africa in 2003, as reported to the pathology-based South African National Cancer Registry [30]; the top five cancers were as follows: cervix (33% [this study] versus 32% [National Cancer Registry]), breast (26% versus 18%), oesophagus (5.2% versus 4.8%), endometrium (3.1% versus 4.1%), and colorectal (2.7% versus 2.1%). As the study was conducted in tertiary referral hospitals, over 90% of cancers in study participants were microscopically verified. In the absence of cancer registries in the participating hospitals, it is not possible to calculate overall response rates. From 5 January 2005 to 31 December 2006 at Charlotte Maxeke Johannesburg Academic Hospital, of 1,853 women approached, 94 women did not participate in the study. The reasons for non-participation were as follows: out of age range (31%), unable to talk/deaf (4%), too ill/dementia (20%), treatment already started (28%), refused (9%), and other reasons (9%).

Oestrogens and progestagens exert different effects on different tissues, and the exact mechanisms underlying their ability to influence the risk of cancer are unclear. Combined oral contraceptives and injectable progestagens exert their main contraceptive effects via the suppression of ovulation, through the feedback inhibition of follicle stimulating hormone and luteinising hormone. Use of combined oral contraceptives suppresses endogenous oestradiol, but this is essentially replaced by exogenous oestradiol and, averaged over a cycle, the net exposure to oestrogen in women taking these preparations may not differ markedly from that in non-users [21]. Use of DMPA results in varying degrees of suppression of oestradiol. While it has been stated that oestradiol levels in users are generally maintained at the early to mid-follicular level [31], other data indicate that among women using DMPA and experiencing amenorrhoea, the majority have oestradiol levels under 100 pmol/l, similar to postmenopausal women [32]. Use also results in very high initial serum levels of progestagen that diminish gradually following administration, which is every 2 mo for norethisterone oenanthate and every 3 mo for DMPA.

Oestrogens are known to increase the rate of cell division within the ductal epithelium of the breast, and hence increase the probability of a mutation occurring or of promotion of an existing mutation; progesterone and progestagens may augment this effect [4],[6],[21]. Data on hormonal therapy for the menopause indicate that, in postmenopausal women, oestrogen in combination with progestagen increases the risk of breast cancer to a much greater extent than oestrogen alone [33],[34]. This suggests an independent effect of progestagens on breast cancer and is consistent with our findings regarding progestagen-only injectable contraceptives. The primary cause of cervical cancer is known to be infection with oncogenic types of the human papillomavirus, but use of combined oral contraceptives appears to act as a co-factor in progression from infection to cervical cancer [35]. Since both combined oral contraceptives and injectable prostagestagen-only contraceptives suppress ovulation and gonadotrophin levels, these are potential explanations for the observed preventative effect on ovarian cancer. Combined oral contraceptives and injectable progestagen-only contraceptives have atrophic and anti-proliferative effects on the endometrium; these effects are believed to underlie their protective effects against endometrial cancer [4],[6],[12],[36].

The net effect on health of hormonal contraceptives includes their intended highly effective contraceptive properties and a range of side effects, some beneficial and some adverse. The evidence from this study, in the context of the evidence to date, indicates that the adverse effects of both oral and injectable hormonal contraceptives on breast and cervical cancer are transient, and risks in users return to those of never users within 10 y after stopping use. However, the exact time point at which the risk in users returns to that in never users is not known. Beneficial effects of both types of hormonal contraceptives on ovarian and endometrial cancers are predominantly in long-duration users.

## Supporting Information

Figure S1 **Sensitivity of cervical cancer main results to the potential confounding effects of Pap smear frequency.** Squares represent ORs, and horizontal lines indicate 95% CI. Diamonds represent the ORs and confidence intervals for the group comprising women from all three exposure categories immediately above.
(TIF)

Figure S2 **Sensitivity of cervical cancer main results to the potential confounding effects of HIV status.** Squares represent ORs, and horizontal lines indicate 95% CI. Diamonds represent the ORs and confidence intervals for the group comprising women from all three exposure categories immediately above.
(TIF)

Figure S3 **Adjusted OR (95% CI) for breast cancer in relation to use of oral and/or injectable contraceptives, demonstrating the effect of removal of single specific cancer types from the control group.** Adjusted for age at diagnosis, year of diagnosis, education, tobacco smoking, alcohol consumption, parity/age at first birth, number of sexual partners, urban/rural residence, and province of birth. Squares represent ORs, and horizontal lines indicate 95% CI.
(TIF)

Figure S4 **Adjusted OR (95% CI) for cervical cancer in relation to use of oral and/or injectable contraceptives, demonstrating the effect of removal of single specific cancer types from the control group.** Adjusted for age at diagnosis, year of diagnosis, education, tobacco smoking, alcohol consumption, parity/age at first birth, number of sexual partners, urban/rural residence, and province of birth. Squares represent ORs, and horizontal lines indicate 95% CI.
(TIF)

Figure S5 **Adjusted OR (95% CI) for ovarian cancer in relation to use of oral and/or injectable contraceptives, demonstrating the effect of removal of single specific cancer types from the control group.** Adjusted for age at diagnosis, year of diagnosis, education, tobacco smoking, alcohol consumption, parity/age at first birth, number of sexual partners, urban/rural residence, and province of birth. Squares represent ORs, and horizontal lines indicate 95% CI.
(TIF)

Figure S6 **Adjusted OR (95% CI) for endometrial cancer in relation to use of oral and/or injectable contraceptives, demonstrating the effect of removal of single specific cancer types from the control group.** Adjusted for age at diagnosis, year of diagnosis, education, tobacco smoking, alcohol consumption, parity/age at first birth, number of sexual partners, urban/rural residence, and province of birth. Squares represent ORs, and horizontal lines indicate 95% CI.
(TIF)

## Abbreviations

DMPA: depot medroxyprogesterone acetate
OR: odds ratio
