# Supplementary figures and images for: Injectable and Oral Contraceptive Use and Cancers of the Breast, Cervix, Ovary, and Endometrium in Black South African Women: Case–Control Study
Source: PLoS Med. 2012 Mar 6;9(3):e1001182. doi: 10.1371/journal.pmed.1001182 (PMC3295825; doi:10.1371/journal.pmed.1001182)

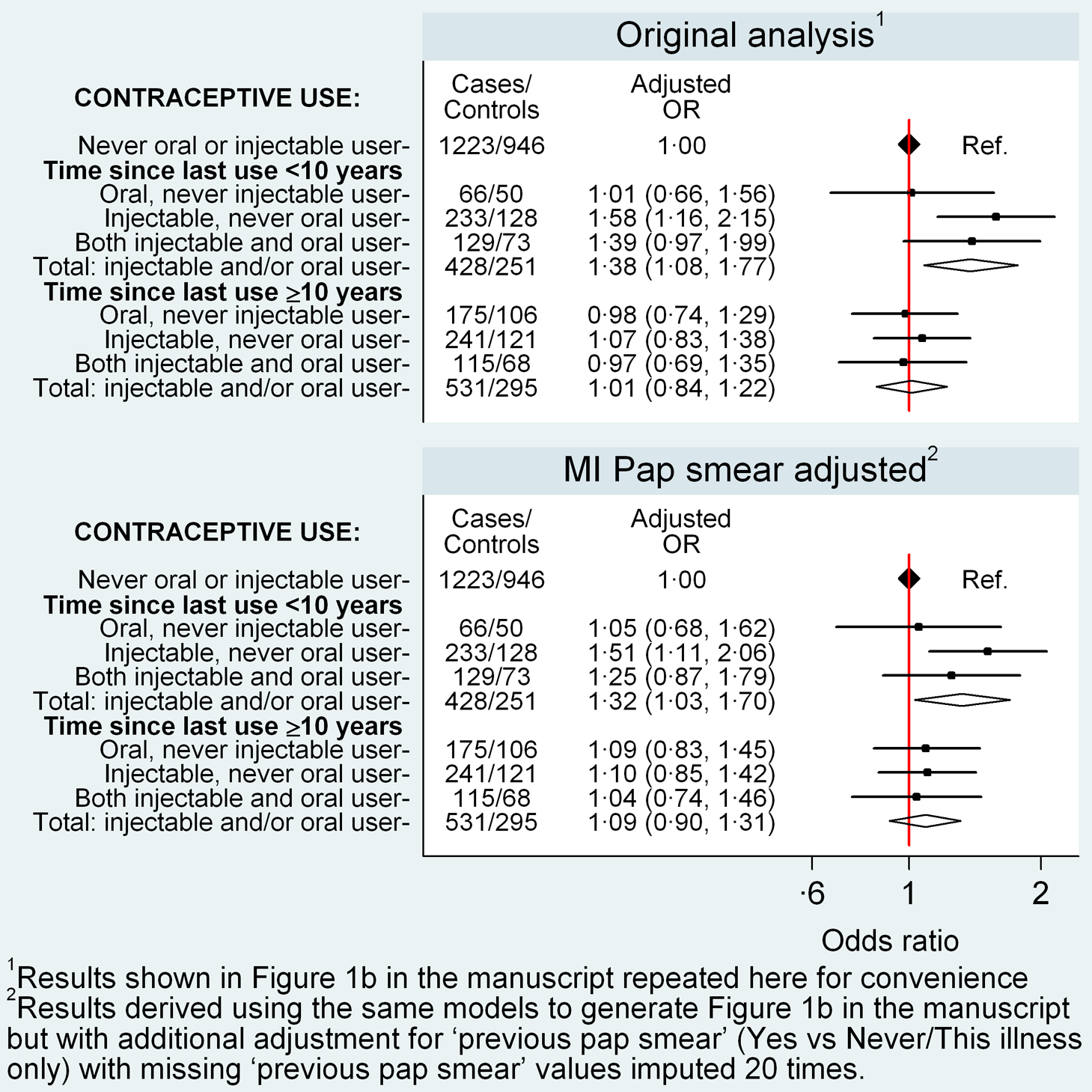

Supplement: Figure S1 — Sensitivity of cervical cancer main results to the potential confounding effects of Pap smear frequency. Squares represent ORs, and horizontal lines indicate 95% CI. Diamonds represent the ORs and confidence intervals for the group comprising women from all three exposure categories immediately above. (TIF) [file pmed.1001182.s001.tif]

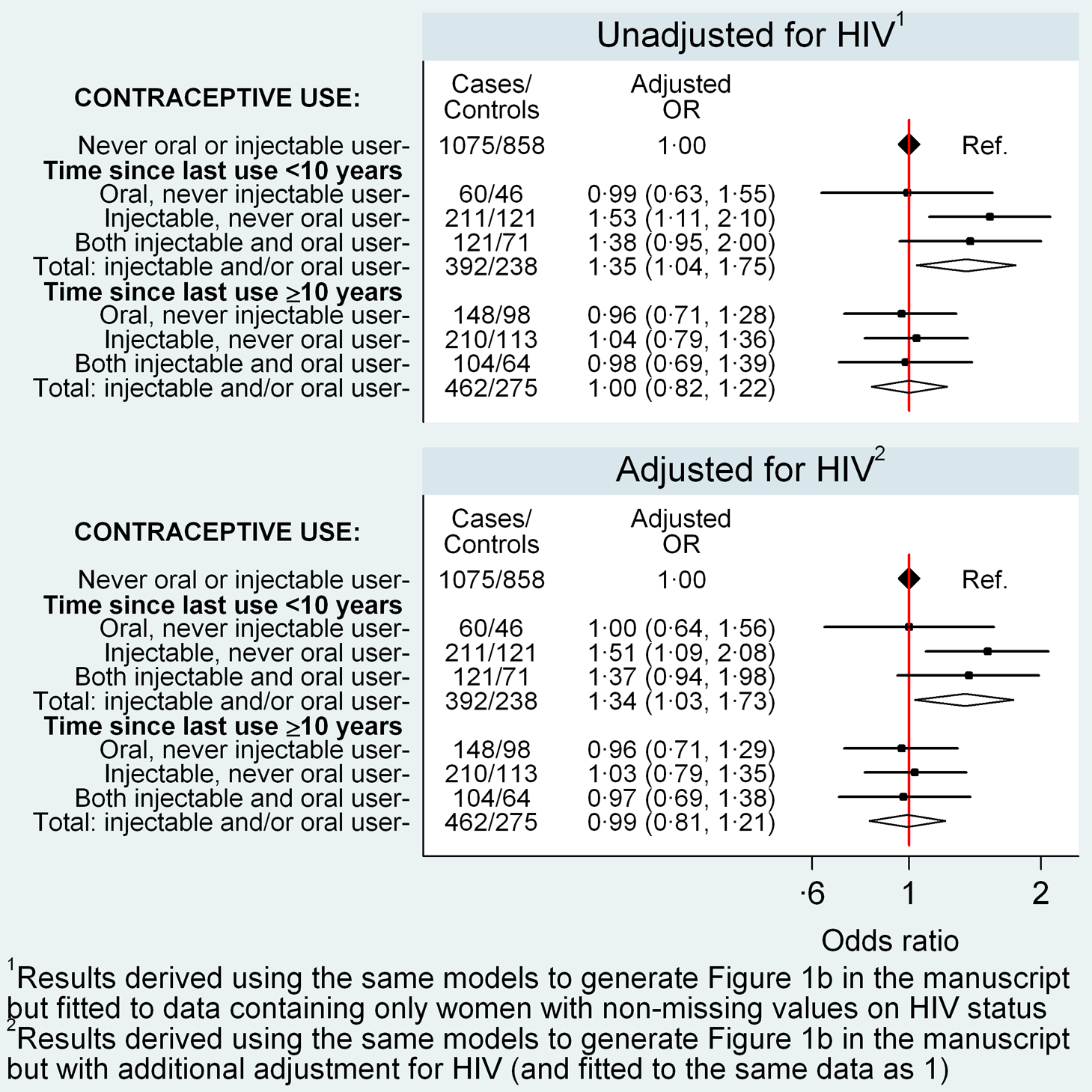

Supplement: Figure S2 — Sensitivity of cervical cancer main results to the potential confounding effects of HIV status. Squares represent ORs, and horizontal lines indicate 95% CI. Diamonds represent the ORs and confidence intervals for the group comprising women from all three exposure categories immediately above. (TIF) [file pmed.1001182.s002.tif]

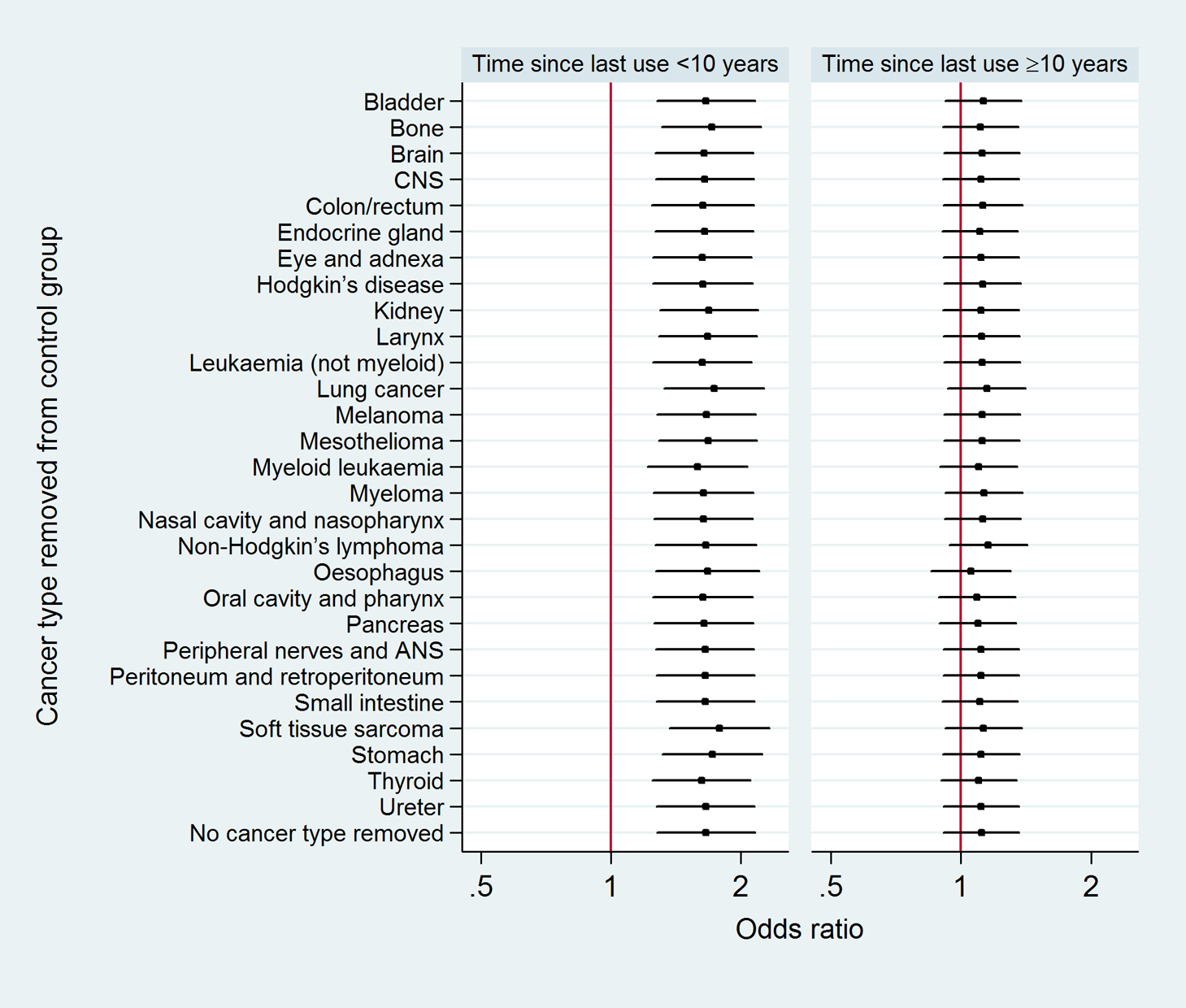

Supplement: Figure S3 — Adjusted OR (95% CI) for breast cancer in relation to use of oral and/or injectable contraceptives, demonstrating the effect of removal of single specific cancer types from the control group. Adjusted for age at diagnosis, year of diagnosis, education, tobacco smoking, alcohol consumption, parity/age at first birth, number of sexual partners, urban/rural residence, and province of birth. Squares represent ORs, and horizontal lines indicate 95% CI. (TIF) [file pmed.1001182.s003.tif]

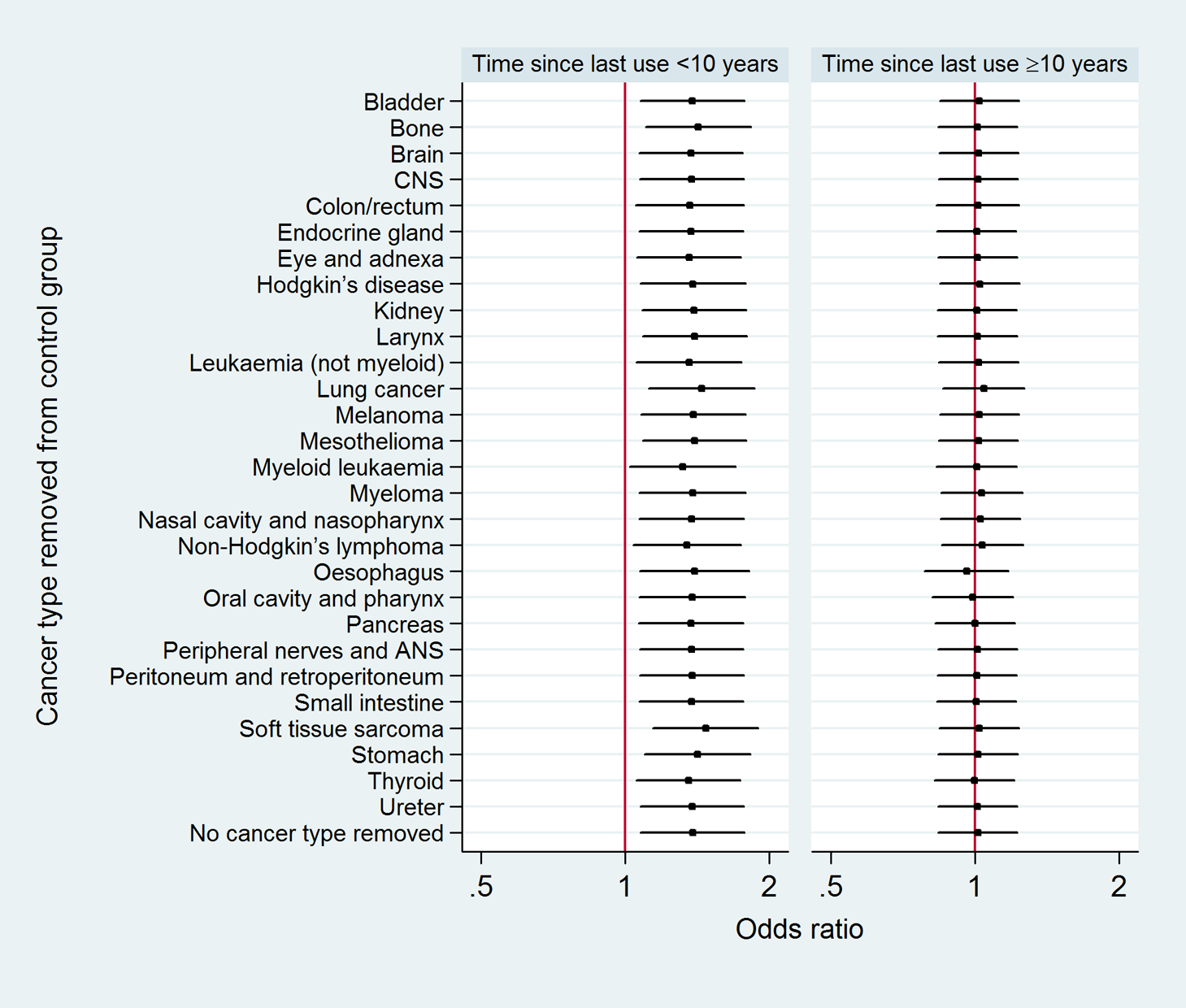

Supplement: Figure S4 — Adjusted OR (95% CI) for cervical cancer in relation to use of oral and/or injectable contraceptives, demonstrating the effect of removal of single specific cancer types from the control group. Adjusted for age at diagnosis, year of diagnosis, education, tobacco smoking, alcohol consumption, parity/age at first birth, number of sexual partners, urban/rural residence, and province of birth. Squares represent ORs, and horizontal lines indicate 95% CI. (TIF) [file pmed.1001182.s004.tif]

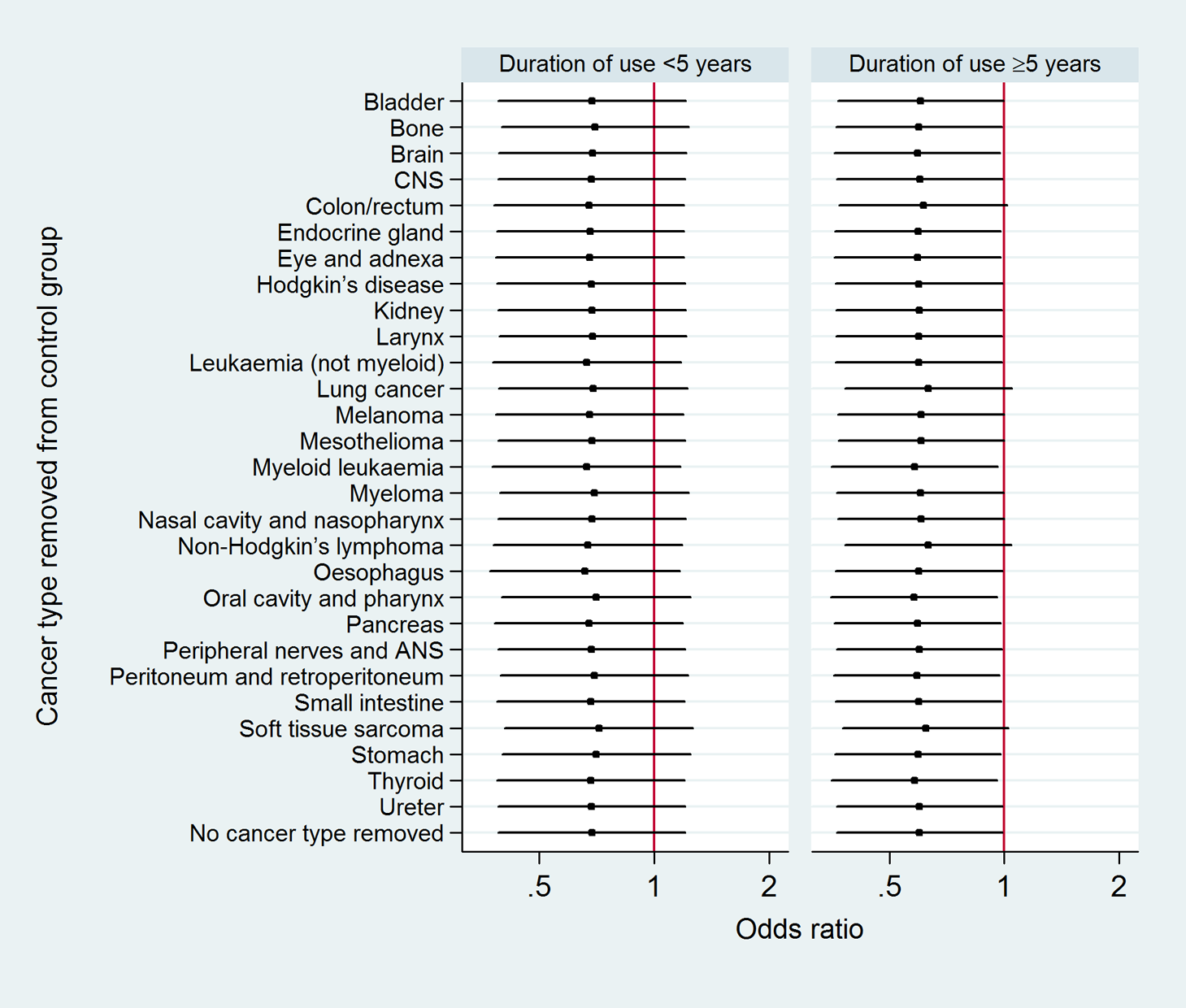

Supplement: Figure S5 — Adjusted OR (95% CI) for ovarian cancer in relation to use of oral and/or injectable contraceptives, demonstrating the effect of removal of single specific cancer types from the control group. Adjusted for age at diagnosis, year of diagnosis, education, tobacco smoking, alcohol consumption, parity/age at first birth, number of sexual partners, urban/rural residence, and province of birth. Squares represent ORs, and horizontal lines indicate 95% CI. (TIF) [file pmed.1001182.s005.tif]

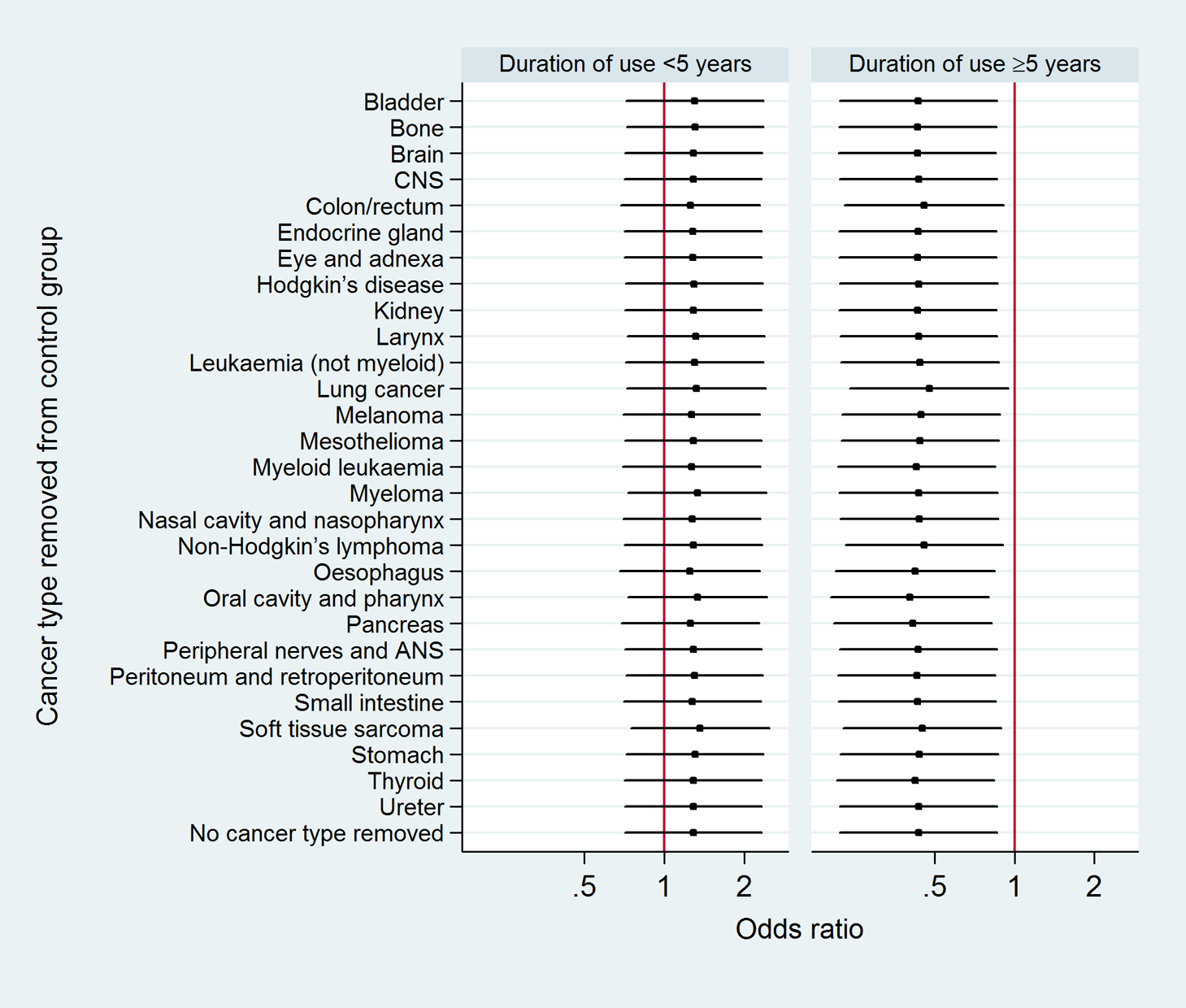

Supplement: Figure S6 — Adjusted OR (95% CI) for endometrial cancer in relation to use of oral and/or injectable contraceptives, demonstrating the effect of removal of single specific cancer types from the control group. Adjusted for age at diagnosis, year of diagnosis, education, tobacco smoking, alcohol consumption, parity/age at first birth, number of sexual partners, urban/rural residence, and province of birth. Squares represent ORs, and horizontal lines indicate 95% CI. (TIF) [file pmed.1001182.s006.tif]
